# Characterization and Public Health Insights of the New Delhi Metallo-β-Lactamase-Producing *Enterobacterales* from Laying Hens in China

**DOI:** 10.3390/microorganisms10040800

**Published:** 2022-04-11

**Authors:** Hongcheng Wei, Linghan Kong, Yulong Wang, Zheren Huang, Xue Yang, Changyu Zhou, Chao Li, Boheng Ma, Cui Li, Changwei Lei, Hongning Wang

**Affiliations:** 1 Key Laboratory of Bio-Resource and Eco-Environment of Ministry of Education, College of Life Sciences, Sichuan University, Chengdu 610065, China; whongcheng@yeah.net (H.W.); m15008448472@163.com (L.K.); shengji2013good@163.com (Y.W.); jeremy96101817@163.com (Z.H.); yangxue970928@163.com (X.Y.); ljl20131077@163.com (C.Z.); lichaomeet@gmail.com (C.L.); bohengm@163.com (B.M.); cui919@126.com (C.L.); leichangwei@126.com (C.L.); 2 Animal Disease Prevention and Food Safety Key Laboratory of Sichuan Province, Chengdu 610065, China

**Keywords:** New Delhi metallo-β-lactamase, *Enterobacterales*, antibiotic-resistance, laying hens, enormous risk

## Abstract

The New Delhi metallo-β-lactamase (NDM) is a major element for the rapid expansion of the carbapenem-resistant *Enterobacterales*, which poses a great challenge to public health security. NDM-producing *Enterobacterales* strains (50 *Escherichia coli*, 40 *Klebsiella pneumoniae*, and 5 *Enterobacter cloacae*) were isolated from laying hens in China for the surveillance of antibiotic-resistant pathogens, and all were found to be multi-drug resistant bacteria. The genomic analysis of these NDM-positive bacteria revealed the ST167, ST617, and ST410 of the fifteen ST-type *E. coli* clones and ST37 of the four ST-type *K. pneumoniae* clones to be the same types as the human-derived strains. Among them, some new clone types were also found. Most of the *bla*_NDM_ genes (*bla*_NDM-1_ or *bla*_NDM-5_) were on the IncX3 plasmids (n = 80) and were distributed in *E. coli*, *K. pneumoniae*, and *E. cloacae*, while the remaining *bla*_NDM-5_ genes were harbored in the *E. coli* ST167 with IncFII plasmids (n = 15). The typeⅠ1 of the eight IncX3 plasmid subtypes was consistent with the human-derived pNDM5_020001 plasmid (accession no. CP032424). In addition, these two plasmids did not affect the growth of the host bacteria and could be reproduced stably without antibiotics. Our study revealed the high genetic propensity of the NDM-positive *Enterobacterales* from the laying hens and human commensal *Enterobacterales*, suggesting the potentially enormous risk of its transmission to humans.

## 1. Introduction

The rapid emergence and extensive dissemination of bacterial antibiotic resistance is a nightmare for humans, with extensively drug-resistant Gram-negative bacteria being a particular focus of attention [1]. Carbapenem antibiotics are the latest generation of β-lactam antibiotics that are commonly used in clinics for treating bacterial infections owing to their high potency and ultra-broad-spectrum antibacterial activity [2,3]. However, the emergence and rapid spread of carbapenem-resistant *Enterobacterales* (CRE), which is in the most serious category of the 12 deadliest drug-resistant bacteria, has emerged as one of the most pressing threats to public health: New Delhi metallo-β-lactamases (NDMs) are the main carbapenem-resistant elements that hydrolyze almost all β-lactam antibiotics and haves been widely distributed worldwide [4,5].

A total of 29 NDM subtypes (NDM-1 to -29) have been identified since NDM1 was first reported in the hospital clinic [5,6]. In addition to NDM-positive bacteria isolated from humans, the animal-derived NDM-carrying bacteria are frequently reported, and traces of NDM have also been found in the retail market as well as in various other environments [7,8]. Although the use of carbapenem antibiotics in poultry breeding has been banned, NDM has been detected in poultry in countries including China, Germany, Egypt, and Pakistan [9,10,11,12]. The transfer and spread of the *bla*_NDM_ genes among Gram-negative bacteria are mainly mediated by plasmids, such as IncX3, IncX4, IncA/C, and IncFII [13]. In addition, *Enterobacterales* is the most convenient carrier of *bla*_NDM_ genes, the isolation rate of carbapenem-resistant *Enterobacterales* (CRE) is increasing rapidly, due to its a large host range and rapid reproduction, and has a trend of global prevalence [13]. NDM-positive *Enterobacterales* generally harbor other antibiotic-resistant genes, showing multi-drug resistance or pan-drug resistance [14,15,16].

China is the world’s largest producer and consumer of eggs, with the world’s largest scale of laying hen farming. This huge population of chickens is mostly reared in large-scale intensive cages. Reports of NDM-positive bacteria in the broiler farms indicate that poultry farms are a potential repository of *bla*_NDM_ genes [9,17]. Carbapenem antibiotics have not been authorized in laying hens. Few studies have focused on the characteristics of the NDM-carrying bacteria in laying hens, and whether they can be transmitted to humans remains unclear. In this research, NDM-positive bacteria were isolated and detected from the laying hens in China and the clonal evolutionary relationship and resistance gene genetic vehicles of NDM-carrying microorganisms from different sources were assessed, indicating the great possibility of the transfer and spread of NDM-positive plasmids of *Enterobacterales* from laying hens to humans.

## 2. Materials and Methods

The animal experiment operations involved in this study were maintained in compliance with the Animal Ethics Committee (AEC) of the College of Life Sciences, Sichuan University (License: SYXK (Chuan) 2013-185).

### 2.1. Sample Collection and Identification

In this study, 1600 samples (200 cloacal swabs per farm) were collected from eight intensive laying hen farms in six provinces (Sichuan n = 2, Shandong n = 2, Anhui n = 1, Hebei n = 1, Hubei n = 1, Gansu n = 1) in China and assigned corresponding numbers (such as SDCRK for Shandong samples, SCCRK for Sichuan samples). All the samples were stored in a refrigerator at 4 °C for identification. All the isolates were cultured in LB broth (37 °C, 180 r/min, 12 h), inoculated into MacConkey medium with 1 μg/mL meropenem and incubated at 37 °C for 16 h, after which the different morphological colonies were picked up from each sample and identified by 16S rRNA sequencing. As mentioned earlier, the carbapenemase-encoding genes *bla*_NDM_ were screened in all the confirmed CRE strains by PCR [18].

### 2.2. Antimicrobial Susceptibility Testing

The antibiotic susceptibility of all *bla*_NDM_-positive isolates was investigated using the broth microdilution method according to the Clinical and Laboratory Standards Institute (CLSI) document M02-A11 (11) and the guidelines of the European Committee on Antimicrobial Susceptibility Testing (EUCAST) (http://www.eucast.org, accessed on 9 May 2019). The antimicrobial agents commonly used in antimicrobial susceptibility testing for human clinic and/or animal husbandry include imipenem (IPM), meropenem (MEM), ceftazidime (CAZ), cefotaxime (CTX), cefoxitin (FOX), gentamicin (GEN), florfenicol (FFC), sulfamethoxazole-trimethoprim (SXT), aztreonam (ATM), ciprofloxacin (CIP), tetracycline (TE), tigecycline (TGC), fosfomycin (FOS), and polymyxin B (PB). The results were interpreted based on the CLSI document M100-S22. The quality control strain used for testing was the *E. coli* American Type Culture Collection (ATCC) 25922.

### 2.3. Whole-Genome Sequencing (WGS) and Identification of the SNPs, STs, and ARGs

The NDM-positive bacteria were inoculated in LB broth and cultured for 8 h at 37 °C with shaking at 180 rpm. The TIANamp Bacteria DNA Kit (Tiangen Biotech Co., Beijing, China, DP302-02) was used to obtain the total DNA according to the instructions. The quality-controlled DNA was processed with the Illumina HiSeq 2000 platform to obtain the original sequence data, and the data were de novo-assembled by SOAPdenovo (http://soap.genomics.org.cn/soapdenovo.html, accessed on 13 November 2019). All the WGS in this study were deposited in the GenBank database under the corresponding accession numbers (Table S3). The WGS of NDM-positive Gram-negative bacteria from the other sources in 2015–2018 were downloaded through the database NCBI. The core-genome SNP-based phylogenetic trees of all the core-genomes were constructed using the software CSI Phylogeny 1.4 and visualized with the software iTOLv5 [19]. MLST was assigned according to the software CGE-MLST 2.0 by mapping data to the alleles using the PubMLST-Public databases and the Institut Pasteur MLST databases. To determine the genetic predisposition of the isolate’s *bla*_NDM_ genes to humans, the genetic correlation between the isolate and the database strain was analyzed. The targeted analysis of all the AMR genes was performed using ResFinder 2.1 [20].

### 2.4. Identification of Plasmids and bla_NDM_ Gene Location and Environment

The determination of the plasmid types was performed using the software Plasmidfinder to analyze the sequencing data [21]. After the splicing and assembly were completed using SPAdes 3.13.0 (University of Manitoba, Manitoba, Canada), NCBI blast and PCR were used to sequence and splice the complete genome sequence of the *bla*_NDM_-plasmid. The ORF annotation of the plasmid was completed using the Sequin software (version 13.70, Bethesda, MD, USA), and the annotated results were submitted to the GenBank database to obtain the accession numbers (Table S4). The genetic environment of the plasmid and *bla*_NDM_ genes were analyzed using the BLAST program (http://blast.ncbi. nlm.nih.gov/Blast.cgi, accessed on 30 June 2020). The plasmid map was drawn using the software Artemis (http://sanger-pathogens.github.io/Artemis/Artemis/, accessed on 17 July 2020). The software Easyfig was used to draw comparative maps of the genetic environment of the different types of plasmids and their *bla*_NDM_ genes.

### 2.5. Conjugation Transfer Experiment

The Rifampicin-resistant *E. coli* EC600 was used as the recipient to study the conjugative transfection of all the types of *bla*_NDM_-IncX3 and *bla*_NDM_-IncFⅡplasmids by filtering and mating, as described previously [22]. The transconjugants were screened with Mueller–Hinton Agar (MHA) plates supplemented with rifampicin (400 µg/mL) and meropenem (8 µg/mL), and the conjugates were further confirmed using disk diffusion susceptibility testing and PCR. The conjugation frequency was determined based on the relative ratio between the transconjugants and the recipients.

### 2.6. Determination of Growth Curves

Transconjugants of eight types of *bla*_NDM_-IncX3 and *bla*_NDM_-IncFII plasmids and the recipient bacteria EC600 were streaked on eosin methylene blue (EMB) agar and cultured overnight at 37 °C. Single colonies were picked up from each culture medium and inoculated in the LB medium before the culture was shaken for 12 h (37 °C, 180 r/min). All the bacterial solutions were adjusted to the same OD_600_ (0.004) using a spectrophotometer (Shanghai Jinghua Technology Instrument Co., Shanghai, China) with sterile LB medium. The adjusted bacterial solution was inoculated into the LB liquid medium (1:1000) and the culture (37 °C, 180 r/min) was shaken, the appropriate amount of culture solution was transferred to the spectrophotometer to determine the OD_600_ every hour. The growth rates were determined from the slope of the growth curve in the logarithmic phase (3–6 h).

### 2.7. Growth Competition Experiment

The paired competitive growth of the transconjugants and recipient bacteria was determined in the LB broth, as mentioned elsewhere [23]. The conjugator bacterial suspension and the recipient bacterial suspension were prepared to the same OD_600_ (0.04) according to the scheme description given above and then mixed at a ratio of 1:1. The mixture was transferred to the LB liquid medium (1:1000) for 6 days of culture (37 °C, 180 r/min), 10-fold serial dilutions were carried out, and the mixture was plated on meropenem (8 µg/mL) and antibiotic-free MHA plates for counting. According to the formula S = ln(CI)/ln(d) (S: selection coefficient; CI: competition index, d: dilution factor), the impact of plasmids on bacterial fitness was assessed. A fitness advantage was judged to exist if S > 0.

### 2.8. The bla_NDM_-Positive Plasmid Stability

The stability of all the types of *bla*_NDM_-positive plasmids was evaluated by subculturing the dilutions (1:1000) of the corresponding transconjugants in antibiotic-free LB medium (37 °C, 180 rpm/min) for 30 days. Bacterial suspensions were diluted and spread on meropenem-containing and non-resistant solid media every 5 days for culture and colony counts, and colonies (n = 10) from both plates were randomly isolated and cultured for disk diffusion sensitivity testing and PCR to verify plasmids and *bla*_NDM_ genes.

### 2.9. Statistical Analysis

Statistical analysis of the growth curves and competition were subjected to a one-way analysis of variance (one-way ANOVA). The homogeneity of the variance was determined using the Levene’s test before an ANOVA was conducted using SPSS Statistics 22.0 (SPSS Inc., Chicago, IL, USA). Graphical representations of the conjugation frequency, growth curves, and stability were drawn using GraphPad Prism 6.0 (GraphPad Software Inc., Broomfield, CO, USA).

## 3. Results

### 3.1. Isolation and Antibiotic-Resistant Phenotype of the bla_NDM_ Gene Carrying Bacteria

**Figure 1:**
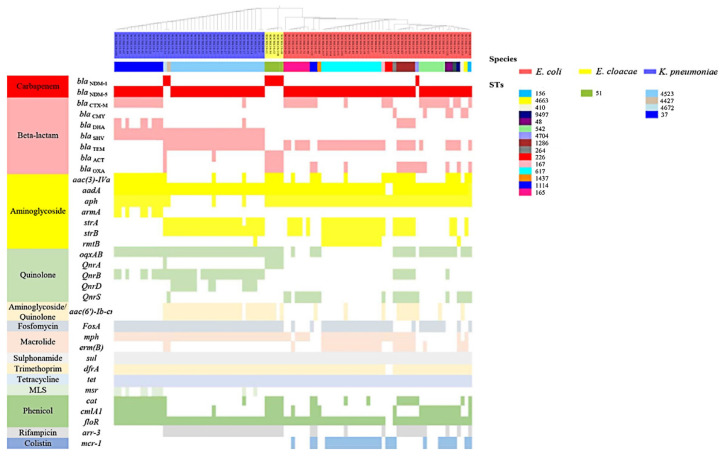
Antibiotic resistance genes (ARGs) and multilocus sequence typing (MLST) clustering of NDM-producing *Enterobacterales*. The species, STs, and antibiotic resistance genes (ARGs) were indicated by different colors.

In this study, 95 strains of the *bla*_NDM_ gene carrying *Enterobacterales* were isolated from four laying hen farms in Sichuan and Shandong, including 50 strains of *E. coli*, 40 strains of *K. pneumoniae*, and 5 strains of *E. cloacae*. No NDM-producing bacteria were detected in the other farms (Figure S1A,B). Of these, one strain of E. coli, two strains of *K. pneumoniae*, and five strains of *E. cloacae* carried the *bla*_NDM-1_ gene and originated from the same farm in Shandong, and the rest carried *bla*_NDM-5_ (Figure 1 and Figure S1C). All the isolated bacteria showed multi-drug resistance to the antibiotics frequently used in clinical settings, including meropenem (MEN) and imipenem (IPM), ceftazidime (CAZ), cefotaxime (CTX), cefoxitin (FOX), sulfamethoxazole-trimethoprim (SXT), and tetracycline (TET), but were susceptible to tigecycline (TGC) (Table 1 and Table S1). *K. pneumoniae* and *E. cloacae* were also not sensitive to florfenicol (FFC) and fosfomycin (FOS); however, they were sensitive to Polymyxin B (PMB), and all the *E. cloacae* were sensitive to aztreonam (ATM), gentamicin (GEN), ciprofloxacin (CIP), and polymyxin B (PMB). Besides, some of the isolated *E. coli* showed resistance to FFC (48/50), CIP (39/50), GEN (30/50), polymyxin B (PB, 29/50), FOS (28/50), and ATM (15/50), and some of *K. pneumoniae* exhibited resistance to CIP (16/40) and GEN (13/40) (Table 1 and Table S1).

### 3.2. Co-Existence of Other Antibiotic Resistance Genes (ARGs) and bla_NDM_ in Isolates

Isolates of the NDM-positive *Enterobacterales* also carried another 32 ARGs (range, 12–32) in 13 other categories (range, 7–13) besides the *bla*_NDM_ gene (Figure 1 and Table S2). The resistance genes *bla*_SHV_, *strA*, *strB*, *fosA*, *mph*, *sul*, *dfrA*, *tet,* and *floR* were detected in *E. coli* and *K. pneumoniae* with a detection rate of greater than 60%, *sul* and *tet* were present in all the isolates, and *fosA* was present in all the *K. pneumoniae*. The *bla*_CMY_ gene (12%, 6/50) of ampC enzyme and the polymyxin resistance gene *mcr-1* (58%, 29/50) were only found in *E. coli*, the aminoglycoside resistance gene *armA* (20%, 8/40), quinolone resistance gene *qnrD* (32.5%, 13/40), and erythromycin resistance gene *msr* (25%, 10/40) were only found in *K. pneumoniae*. The β-lactam resistance gene *bla*_DHA_ (10%, 5/50) existed in *E. coli*, which was only observed in ST1286 strains, the aminoglycoside resistance gene *rmtB* was mainly found in the ST617 *E. coli* (94.1%, 16/17), and the quinolone resistance gene *qnrB* was mainly found in the ST1286 *E. coli* (83.3%, 5/6).

### 3.3. Genomic Diversity Characteristics of the NDM-Positive Enterobacterales

**Figure 2:**
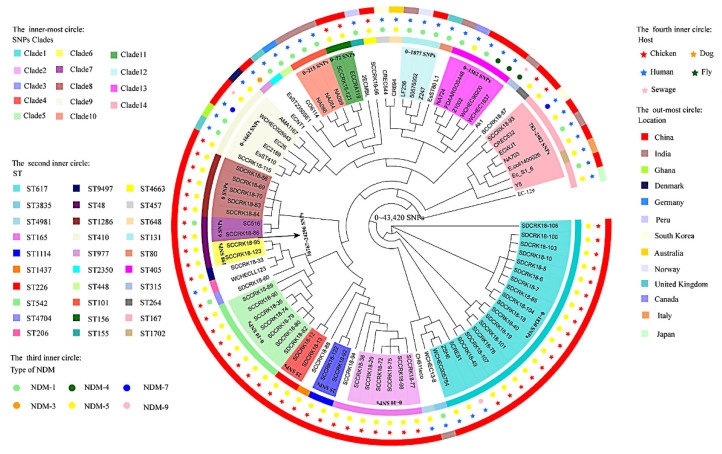
Core genome evolution relationship of the *bla*_NDM_ gene carrying *E. coli* strains (50 strains from this study, 39 strains from the database). The genomic sequence of *E. coli* K-12 (accession number CP025268) was selected as a reference genome. The SNPs, STs, NDM, host, and country were indicated by different colors and shapes.

Isolates of the NDM-positive *E. coli* were clustered into 15 distinct sequence types (STs) via silico multilocus sequence typing (MLST), including a new type of ST 264 (1/50). The majority of the isolates were concentrated into ST617 (16/50), ST165 (7/50), ST542 (7/50), and ST1286 (5/50) (Figure 1 and Figure S2A). The comparative analysis of the WGS-single nucleotide polymorphisms (SNPs) of *E. coli* in this experiment and the NDM-positive *E. coli* from different sources in the database (https://www.ncbi.nlm.nih.gov, accessed on 13 August 2020, n = 39) showed that the bacterial clones ST167, ST617, and ST410 were identified in our samples and humans in different countries or regions, but the SNPs of the same ST-type strains from different sources were far apart (Figure 2). In this study, the ST167 and ST410 isolates were only distributed in Sichuan, and the ST617 strain was found in Shandong and Sichuan. Although the ST542 strains were isolated from two different regions, they only differed by 0–10 SNPs, the ST165 and ST114 strains were from Sichuan, with differences of 0–10 SNP and 25 SNP, respectively. The ST1286 and ST226 strains were isolated from the same farm in Shandong, with differences of 0 SNP and 25 SNPs, respectively, while the ST48 strain SCCRK-86 from Sichuan differed by only 6 SNPs from the isotype SC516 strain (accession no. NZ_CP025048.1) in the database. The ST156 strains from Sichuan and the ECCRA119 strain (accession no. CP029242) in the database also differed by only 72 SNPs (Figure 2).

**Figure 3:**
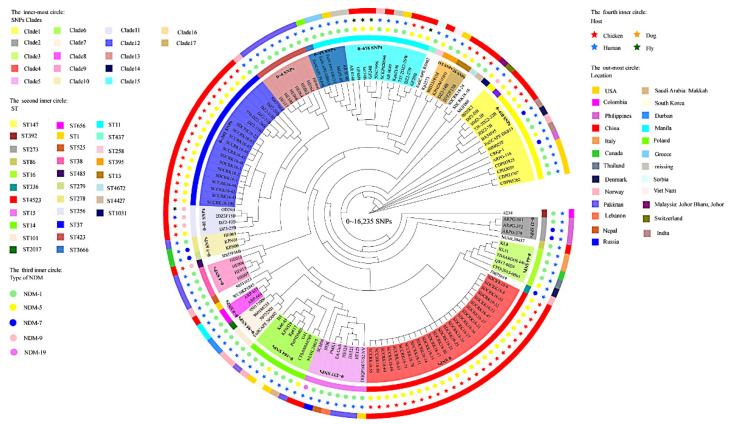
Core genome evolution relationship of the *bla*_NDM_ gene carrying *K. pneumoniae* strains (40 strains from this study, 97 strains from the database). The genomic sequence of *K. Pneumoniae* KCTC2242 (accession number CP002910) was selected as a reference genome. The SNPs, STs, NDM, host, and country were indicated by different colors and shapes.

Isolates of the NDM-positive *K. pneumoniae* were clustered into 4 STs, including ST37 (13/40), ST4523 (25/40), and two new types of ST4427 (1/40) and ST4672 (1/40) (Figure 1 and Figure S2B). Although the ST37 and ST4523 strains were distributed across different regions, there were no differences in the SNP of ST4523 strains, and only 0–25 SNPs difference among the ST37 strains (Figure 3). It is particularly noteworthy that the comparison of the genomes of the strains from this experiment and the *bla*_NDM_-carrying *K. pneumoniae* from the database (https://www.ncbi.nlm.nih.gov, accessed on 16 August 2020, n = 97) revealed that the ST37 isolate from this study differed from the human isotype strain by 156 SNPs.

All the *E. cloacae* strains were clustered into ST51 and distributed across the same farm in Shandong (Figure 1). No comparative analysis was conducted due to relatively few strains and single information.

### 3.4. bla_NDM_-Harboring Plasmids and Genetic Context of bla_NDM_

All the isolates harbored two or more types of plasmid replicons (range, 2–10): seventeen, eight, and two types of plasmid replicons were detected in the NDM-producing *E. coli*, *K. pneumoniae*, and *E. cloacae* isolates, respectively (Table S2). All the *bla*_NDM-1_ (8/95) and most of the *bla*_NDM-5_ (72/95) were located on the IncX3 plasmids across 8 subtypes, and the remaining *bla*_NDM-5_ (15/95) were located on the IncFII plasmid (Figure S3 and Table S2).

**Figure 4:**
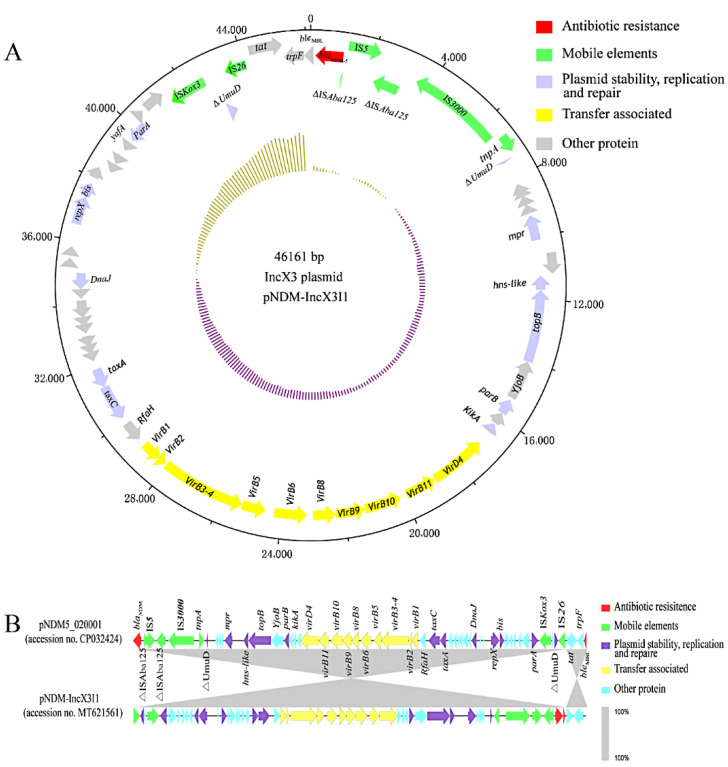
Schematic map of the IncX3 plasmid and genetic environment of *bla*_NDM_ genes. Regions of ≥99.0% nucleotide sequence identity were shaded in gray. (**A**) Genetic environment of pNDM-IncX3I1. (**B**) Comparative genomics of pSDCRK-IncFNDM5 and pNDM5_020001.

The skeleton structure of all the IncX3 plasmids was highly conserved with the same △*UmuD* genetic structure (△*UmuD-IS26*, IS*3000-tnpA-*△*UmuD*). Mutations of different subtypes mainly occurred in the region between the two △*UmuD* (Figure S3). The plasmid of type I1 (pNDM-IncX3I1, 10/80) was 46,161 bp with a 47% G+C content, and 100% nucleotide identity with pNDM5_020001 (accession no. CP032424), a 46,161bp IncX3 plasmid identified in an *E. coli* strain from the West China Hospital in China (Figure 4A,B).

**Figure 5:**
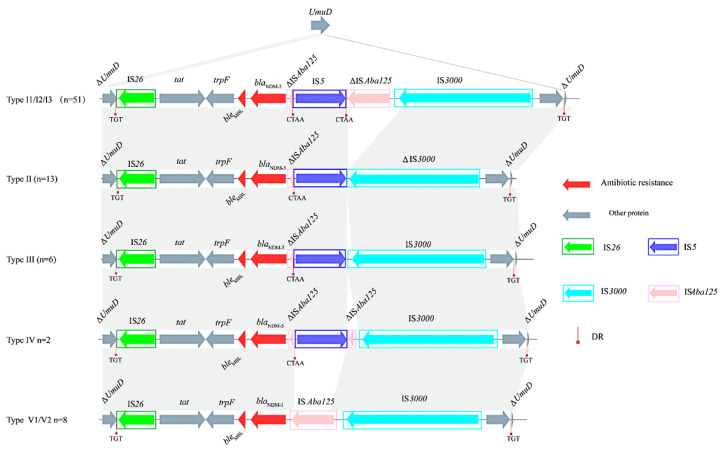
Comparison of the genomic genetic environment of *bla*_NDM_ genes in IncX3. The arrows marked the direction of gene transcription, and the gene categories were differentiated by colors. The homologous regions were marked by grey shading.

*bla*_NDM-5_ was adjacent to a transferable element IS*Aba125* divided by IS*5* in pNDM-IncX3I1/ I2/ I3, orf and IS*3000* were upstream of *bla*_NDM-5_, and downstream of *bla*_NDM-5_ were *ble*_MBL_ (mediating bleomycin resistance), *trpF* (encoding an N-5′-phosphoribosyl anthranilate isomerase), *tat* (encoding a twin-arginine translocation pathway signal sequence domain protein), and an IS*26* (Figure 5). Unlike pNDM-IncX3I1, there was only one point mutation in the type I2 (pNDM-IncX3I2, 25/80) and type I3 (pNDM-IncX3I3, 17/80) blaNDM-IncX3 plasmid (Table 2). Comparison with pNDM-IncX3I1 revealed a series of deletion mutations present between *bla*_NDM-5_ and IS*3000* in the blaNDM-IncX3 plasmid of Type II (pNDM-IncX3II, 13/80), Type III (pNDM-IncX3III, 5/80), Type IV (pNDM-IncX3IV, 2/80), Type V1 (pNDM-IncX3V1, 1/80), and Type V2 (pNDM-IncX3V2, 7/80) (Figure 5). Type I1, TypeⅠ3, Type III, and TypeⅤ1 IncX3 plasmids were only found in *E. coli*, while TypeⅡ and TypeⅣ IncX3 plasmids were only found in *K. pneumoniae* (Figure S4). In the plasmid of pNDM-IncX3 II, except for one point mutation, IS*Aba125* in the downstream of IS*5* and part of the 3′ end of IS*3000* were deleted. The deletion of △IS*Aba125* downstream of IS*5* was found in pNDM-IncX3III. In the plasmid of pNDM-IncX3IV, the downstream of IS*5* retained the residues of △IS*Aba125*. The complete IS*Aba125* was observed in pNDM-IncX3V1/ V2, no IS*5* was found and there were five points mutations in the plasmid of pNDM- IncX3V1/V2 instead of pNDM-IncX3I1 (Figure 5 and Table 2).

**Figure 6:**
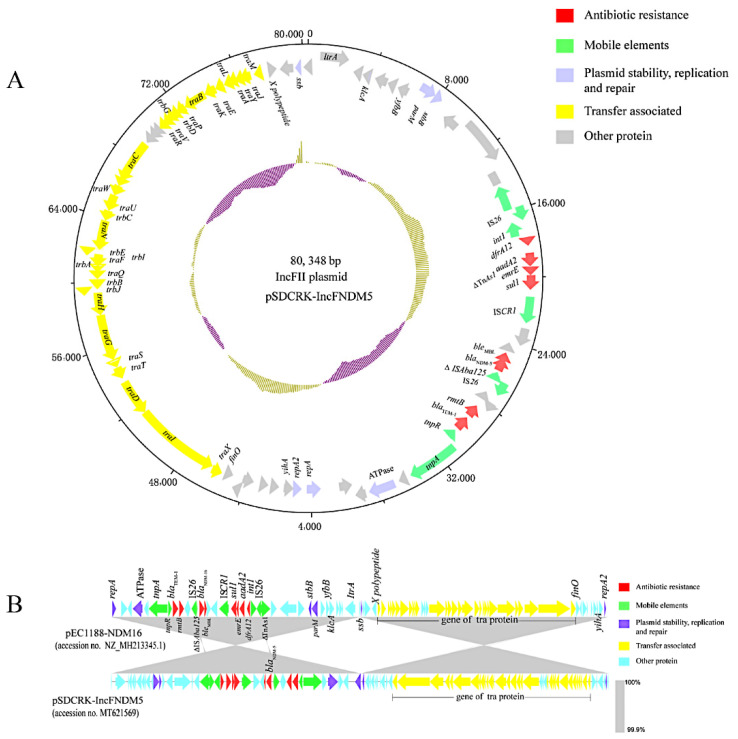
Schematic map of blaNDM-IncFII plasmid. Regions with ≥99.0% nucleotide sequence identity were shaded in gray. (**A**) Genetic environment of pSDCRK-IncFNDM5. (**B**) Comparative genomics of pSDCRK-IncFNDM5 and pEC1188-NDM16.

All the *bla*_NDM_-IncFII plasmids were stored in ST617 *E. coli* with a 100% nucleotide identity and designated as pSDCDK-IncFNDM5, which was 80,348 bp with a 54% G + C content and encoded *bla*_NDM-5_ as well as seven other ARGs, including *bla*_TEM-1_ (β-lactam resistance), *emrE* (erythromycin resistance), *rmtB*, *sul1,* and *aadA2* (aminoglycoside resistance), *ble*_MBL_ (bleomycin resistance), and *dfrA12* (trimethoprim resistance) (Figure 6A). All the ARGs were encapsulated in a box between two IS*26*, IS*26*-△IS*Aba125* was upstream of *bla*_NDM-5_ in pSDCDK-IncFNDM5, downstream of *bla*_NDM-5_ were *ble*_MBL_, *trpF*, *tat,* and an IS*CR1*, which constituted an “IS*26*-IS*CR1*” transposon (IS*26*-△IS*Aba125*-*bla*_NDM_-*ble*_MBL_-*tat*-*trpF*-IS*CR1*). The genetic context of *bla*_NDM-5_ in pSDCDK-IncFNDM5 was highly similar (99.9% overall nucleotide identity and 100% query coverage) to that of the *bla*_NDM_-16-positive IncFII plasmid pEC1188-NDM16 (accession no. NZ_MH213345.1). An 80,215 bp IncFII plasmid was identified in an *E. coli* strain from the Zhejiang Provincial People’s Hospital in China (Figure 6B). The structure of the multi-drug resistance zone outside the *bla*_NDM_ gene of the two plasmids was the same, where the plasmid skeleton region of pEC1188-NDM16 which was 130 bp in length was missing from pSDCDK-IncFNDM5 (38710–38849), the fragment was located in the upstream non-coding region of the replication gene *repA*.

### 3.5. Fitness and Genetic Stability of the bla_NDM_-Carrying Plasmids

**Figure 7:**
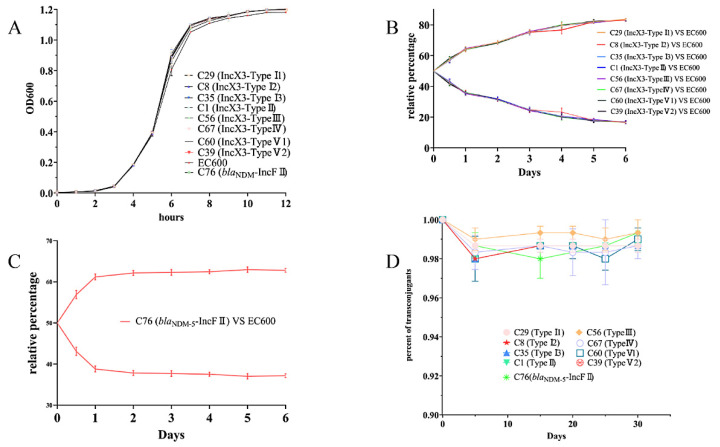
Adaptability and stability of *bla*_NDM_-positive plasmid. (**A**) Growth curve of 9 kinds of transconjugant. (**B**) Competitive growth results obtained for *bla*_NDM_-IncX3 transconjugants and recipient bacteria EC600. (**C**) Competitive growth results obtained for carrying *bla*_NDM-5_-IncFⅡ transconjugants and recipient bacteria EC600. (**D**) Results obtained for continuous passage of *bla*_NDM_-IncX3 and *bla*_NDM_-IncFⅡ-carrying plasmid transconjugants.

The conjugation of the *bla*_NDM_-IncFII plasmid was not as active as that of the *bla*_NDM_-IncX3 plasmids (Figure S5). No effect on growth was observed between the transconjugants of the *bla*_NDM_-carrying plasmids and the recipient strain EC600 (Figure 7A). The growth trend of the conjugants was better than that of the recipient bacteria, the IncX3-TypeI1 and IncX3-TypeI3-harbouring conjugants had the highest growth rate, and the growth rate of the transconjugants of the IncX3 plasmids was higher than that of the IncFⅡ plasmid (Figure S6). Competitive assays of the plasmid fitness revealed that the ratios of IncX3 plasmid-carrying bacteria continued to increase in the co-culture of transconjugants and recipient bacteria over a period of 6 days (range, 83.20–83.67%) (Figure 7B). The relative concentration increased (33.67%), and the growth selection coefficient (0.0244) of the IncX3-TypeI1-harbouring conjugants increased the most, corresponding to the low relative concentration increase in (33.20%), and the selection coefficient of (0.0231), the IncX3-TypeV2-harbouring conjugants (Figure 7B and Figure S7A). The relative concentration of the transconjugant of the IncFⅡ plasmid increased significantly in 0–1 day, and there was no alteration in the later period (about 62%). The growth selection coefficient of the IncFⅡ-carrying conjugants was 0.0032 (Figure 7C and Figure S7B). The results obtained for the continuous subculture of the plasmid-carrying transconjugants in the antibiotic-free conditions demonstrated that all types of resistant plasmids could stably exist in the host bacteria and that the proportion of all types of plasmid-carrying bacteria remained above 98% in the 30-day antibiotic-free culture process (Figure 7D).

## 4. Discussion

Similar to in human hospital clinics, *Enterobacterales species* are also the major NDM-producing bacteria in layer hen farms [13]. *bla*_NDM-5_ was an NDM variant and had an overwhelming epidemic dominance among the tested farms, bearing a resemblance to the previous NDM survey results of the broiler farms [14]. Only a few *bla*_NDM-1_-carrying strains were obtained in one farm, which was different from the main prevalence of both *bla*_NDM-1_ and *bla*_NDM-5_ seen in human clinics [24]. Although different reports have shown that there are deviations in the isolation rate of various mutants of the *bla*_NDM_ gene from poultry [9,17], the high prevalence of *bla*_NDM-5_ indicated its widespread distribution and spread in the poultry and farm environments. Isolates of the NDM-positive bacteria have been detected to have multi-drug resistance, meaning they were inseparable from the coexistence of multiple-drug-resistant genes. The co-selection of these coexisting resistance genes may be one reason why the *bla*_NDM_ genes still existed in carbapenem-free farms. Polymyxin is one of the limited means by which to treat serious infections caused by *bla*_NDM_-carrying Gram-negative bacteria. Contrary to a previous monitoring report in nationwide clinics [25], in our study, *E. coli* was found to be co-resistant to polymyxin and carbapenem antibiotics, but not to *K. pneumoniae* and *E. cloacae*. The emergence and spread of multi-drug-resistant *Enterobacterales* seriously threatens the health of humans and animals.

The clonal diversity of the NDM-positive *E. coli* has been frequently reported, indicating that the prevalence of *bla*_NDM_ in *E. coli* was not only caused by clonal propagation [26,27]. The distribution of *E. coli* clone types differs in different regions or hosts, in this study, the *E. coli* cloning types ST167, ST167, and ST410 have been reported in humans, sewage, and retail markets [13,28,29]. This suggested that the clonal types of NDM-producing *E. coli* isolates that were ubiquitous in multiple hosts and in different environments deserve more attention. In this study, the ST206 type cloned strain isolated from Sichuan and the chicken-derived isotype strain in the same region isolated in 2015 only differed by six SNPs. There was a slight difference in the Sichuan ST156 type strain cloned in this study and the poultry-derived isotype strain collected in Zhejiang in 2017 [30]. This suggested that certain clonal types of NDM-positive *E. coli* had long-term clonal transmission in domestic farms.

Moreover, the result of our study confirmed the previous report that ST37 was the dominant *K. pneumoniae* clone type in Shandong farms [17]. Simultaneously, ST37 has also been frequently reported as the dominant clone of the NDM-positive *K. pneumoniae* in human clinics [31,32], which has even been found to persist in the environment and spread between animals and humans [33]. The SNPs of ST37 *K. pneumoniae* isolated by us and the same type of *K. pneumoniae* in humans were less different. It is necessary to strengthen standardized management in animal breeding to avoid this type of NDM-positive bacteria spreading between humans and animals. In addition, no SNP differences in ST4523 *K. pneumoniae* were found between farms in two regions without a direct relation, suggesting that its distribution and spread should be kept under constant surveillance.

The IncX3 plasmid was the most important transmission vector of the *bla*_NDM_ genes, and frequently found in clinics, farms, food chains, and the environment [17,33,34]. The presence of the IncX3 plasmid in all ST types of the three bacterial species was substantiated by a previous report on the broad-spectrum host range of IncX3 [32]. Although the use of carbapenem in animal feed has been forbidden, its diversified sources and wide host spectrum have been accountable for the large-scale spread of IncX3 plasmid carrying the *bla*_NDM_ gene in various environments. In this study, pNDM-IncX3I1 was found to have a 100% homology with the human clinical plasmid pNDM5_020001, indicating that the *bla*_NDM_-IncX3 plasmid has the great potential for cross-region and cross-species transmission. Seven other subtypes of IncX3 were also identified, and these mutations all occurred in the insertion sequence IS*Aba125*, IS*5*, and IS*3000* between the IS*3000* and *bla*_NDM_ gene, and deletions or missing mutations of these inserted sequences have been reported in the *bla*_NDM_-IncX3 plasmids collected from human clinics and retail meat in different regions [35,36]. This suggested that the sequence region between the IS*3000* and *bla*_NDM_ gene was the most common mutation-prone region of *bla*_NDM_-IncX3 plasmids.

Another universal *bla*_NDM_ gene carrier IncFⅡ plasmid was found to have a larger genome than the IncX3 plasmid, and to often carry multiple drug-resistant genes [37,38]. Our observation exhibited that the pSDCDK-IncFNDM5 plasmid contained seven coexisting ARGs except for the *bla*_NDM-5_ gene. The structures of the backbone region and multi-drug resistance zone outside the *bla*_NDM_ gene of the pSDCDK-IncFNDM5 plasmid were highly consistent with those of a pEC1188-NDM16 plasmid obtained from a human clinic [39]. This revealed the potential for the cross-species transmission of the *bla*_NDM_-IncFⅡ plasmids. The adhesion of the *bla*_NDM_ genes of the above two plasmids to the composite transposon “IS*26*-IS*CR1*” (IS*26*-△IS*Aba125*-*bla*_NDM_-*ble*_MBL_-*tat*-*trpF*-IS*CR1*) might be an important reason for transforming *bla*_NDM_ genes, which was different from that of a previous report which showed that the two IS*CR1*-entrained NDM genes (IS*CR1*-△*dsbc*-*trpF*-*ble*-*bla*_NDM_-*qacE*△*1*-*sul1*-IS*CR1*) were integrated into the IncHI5 plasmid through circularization and recombination [40]. However, this mechanism deserved further study.

Furthermore, evidence has revealed that plasmids carry *bla*_NDM_ genes for large-scale and long-term reproduction. The results were found to be consistent with those of previous reports [41,42], and it was found that all the types of *bla*_NDM_-IncX3 plasmid identified in this study failed to affect the growth of the host bacteria, even conferring a certain fitness advantage to the host bacteria. Similarly, the *bla*_NDM_-IncFⅡ plasmid did not affect the growth of the host bacteria but imparted the host bacteria with a fitness not as intense as that of the IncX3 plasmid. To add to these worries, not only do all types of *bla*_NDM_-harboring plasmids have no cost of adaptability, but they can also reproduce stably in the host bacteria without antibiotic selection pressure. This could provide a partial explanation of the reason for the *bla*_NDM_ genes to distribute and reproduce stably in the carbapenem-free farms with its harboring plasmid.

## 5. Conclusions

NDM-positive multi-drug resistant *Enterobacterales* were isolated from laying hens, leading to the identification of some novel clone types and several clone types that also exist in humans and the environment. Among the eight *bla*_NDM_-IncX3 plasmid subtypes and the *bla*_NDM_-IncFⅡ plasmid, some were found to be identical or highly homologous to human-derived plasmids, and all types of plasmids were found to confer a certain fitness advantage to the host bacteria enabling them to reproduce stably without antibiotic selection. These results indicated that the high genetic propensity of the NDM-positive plasmids of poultry-derived *Enterobacterales* for spreading into humans deserved serious attention and also suggested the importance of continuous surveillance for the prevalence of NDM-positive bacteria in poultry to avoid public health incidents.

## Figures and Tables

**Table 1:** Results for antibiotic resistance rate of the *bla*_NDM_ gene carrying *Enterobacterales*.

Antibiotics	Number of Resistant Isolates			Resistance Rate (%)		
	*E. coli*	*K. peneumoniae*	*E. cloacae*	*E. coli*	*K. peneumoniae*	*E. cloacae*
MEN	50	40	5	100	100	100
IPM	50	40	5	100	100	100
CAZ	50	40	5	100	100	100
CTX	50	40	5	100	100	100
FOX	50	40	5	100	100	100
SXT	50	40	5	100	100	100
TET	50	40	5	100	100	100
ATM	15	1	0	30	2.5	0
TGC	0	0	0	0	0	0
GEN	30	13	0	60	32.5	0
CIP	39	16	0	78	40	0
FOS	28	40	5	56	100	100
PB	29	0	0	58	0	0
FFC	48	40	5	96	100	100

**Table 2:** Gene mutation sites of blaNDM-IncX3 plasmids *.

	Type I1	Type I2	Type I3	Type II	Type III	Type IV	Type V1	Type V2	Location
2325 (C-T)	−	−	−	−	−	−	+	+	IS*5*
10232 (A-G)	−	−	−	−	−	−	+	+	non-coding region
12470 (C-T)	−	−	−	−	−	−	+	−	*topB*
13038 (G-A)	−	−	−	−	−	−	−	+	*topB*
13627 (A-G)	−	−	+	−	−	−	−	−	*topB*
19367 (G-T)	−	−	−	−	−	−	−	+	*virB11*
21112 (A-G)	−	+	−	−	−	−	−	−	*virB10*
25945 (C-T)	−	−	−	−	−	+	−	−	*virB3-4*
26374 (C-A)	−	−	−	−	−	−	+	−	*virB3-4*
35556 (G-A)	−	−	−	−	−	−	+	+	non-coding region
36080 (T-C)	−	−	−	+	−	−	−	−	non-coding region
NO. Strains	9	25	17	13	6	2	1	7	—

* “+”, stands for mutation; “−”, means no mutation.

## Data Availability

The genome sequences of the isolates in this study can be retrieved and downloaded from the NCBI database (https://www.ncbi.nlm.nih.gov/, accessed on 27 June 2020) according to the accession numbers in Table S3.

## Supplementary Materials

The following supporting information can be downloaded at: https://www.mdpi.com/article/10.3390/microorganisms10040800/s1, Figure S1: Distribution of NDM-producing *Enterobacterales*, sample collection diagram of large-scale chicken farms (A), species identification and distribution results of *bla*_NDM_-carrying *Enterobacterales* (B), type identification and distribution results of *bla*_NDM_ genes (C). Figure S2: Phylogenetic trees of NDM-producing *Enterobacterales*, phylogenetic trees of 50 NDM-producing *E. coli* (A), phylogenetic trees of 40 NDM-producing *K. pneumoniae* (B). Figure S3: Comparison of genetic structure of *bla*_NDM_-IncX3 plasmids. Figure S4: Distribution of 9 kinds of *bla*_NDM_-plasmids among 95 Gram-negative bacteria. Figure S5: Transconjugative frequencies of *bla*_NDM_-IncX3 and *bla*_NDM_-IncFⅡ plasmids. Figure S6: Growth rate of 9 kinds of transconjugants. Figure S7: Selection coefficient of 9 kinds of transconjugants. Table S1: Antibiotic resistance phenotypes of the isolates in this study. Table S2: MLST typing and plasmids of the isolates in this study. Table S3: NCBI accession numbers of 95 *bla*_NDM_ gene carrying Gram-negative bacteria. Table S4: GeneBank accession numbers of *bla*_NDM_ gene carrying plasmids.
