# Supplementary material for: Characterization and Public Health Insights of the New Delhi Metallo-β-Lactamase-Producing Enterobacterales from Laying Hens in China
Source: Microorganisms. 2022 Apr 11;10(4):800. doi: 10.3390/microorganisms10040800 (PMC9029685; doi:10.3390/microorganisms10040800)
Supplement: Supplementary file 1 [file microorganisms-10-00800-s001.zip › Supplemental_Figures.pdf]

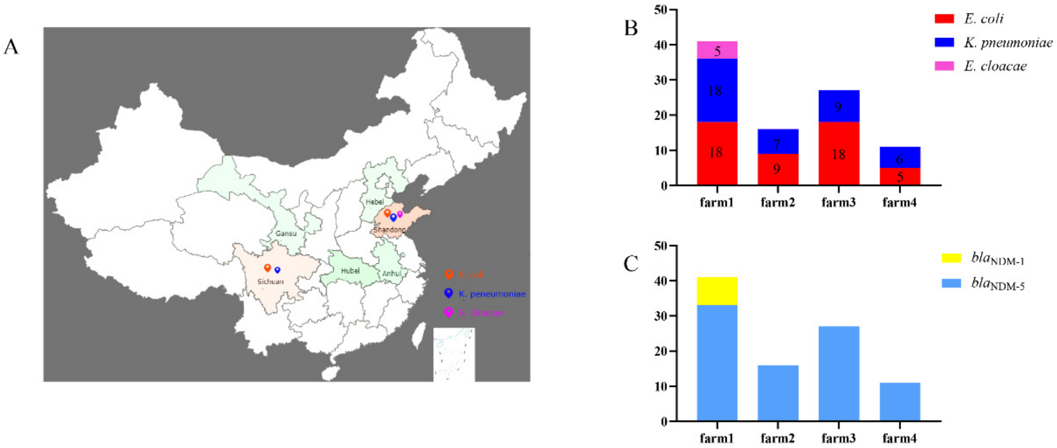

Figure S1. Distribution of NDM-producing *Enterobacteriaceae*. (A) Sample collection diagram of large-scale chicken farms. (B) Species identification and distribution results of *bla*NDM-carrying *Enterobacteriaceae*. (C) Type identification and distribution results of *bla*NDM genes.

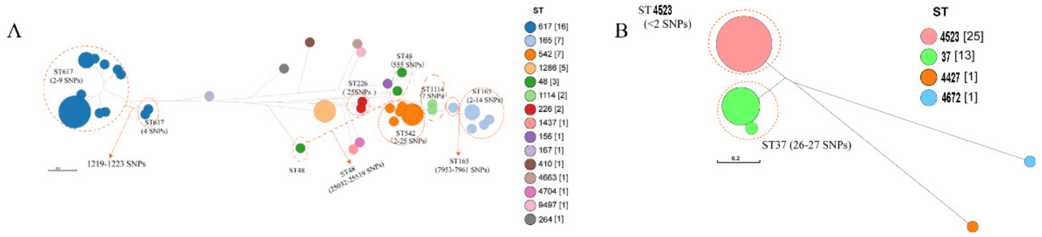

Figure S2. Phylogenetic trees of NDM-producing *Enterobacteriaceae*. (A) Phylogenetic trees of 50 NDM-producing *E. coli*. (B) Phylogenetic trees of 40 NDM-producing *K. pneumoniae*.

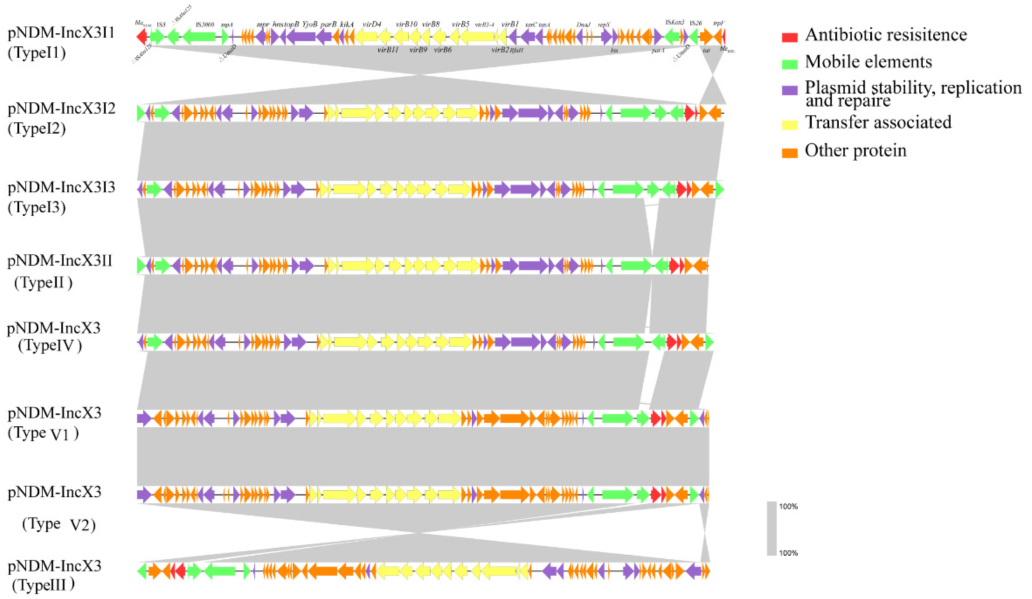

Figure S3. Comparison of genetic structure of *bla*NDM-IncX3 plasmids. The arrows marked the direction of gene transcription, and the gene categories were differentiated by colors. the homologous regions were marked by grey shading.

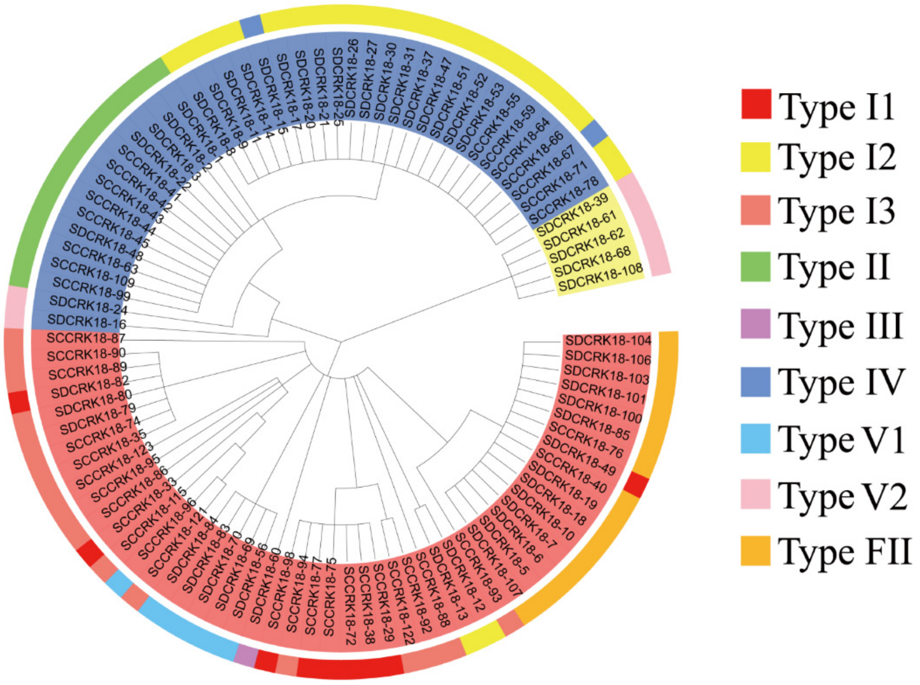

Figure S4. Distribution of 9 kinds of *bla*NDM-plasmids among 95 Gram negative bacteria. Species and plasmids were indicated by different colors.

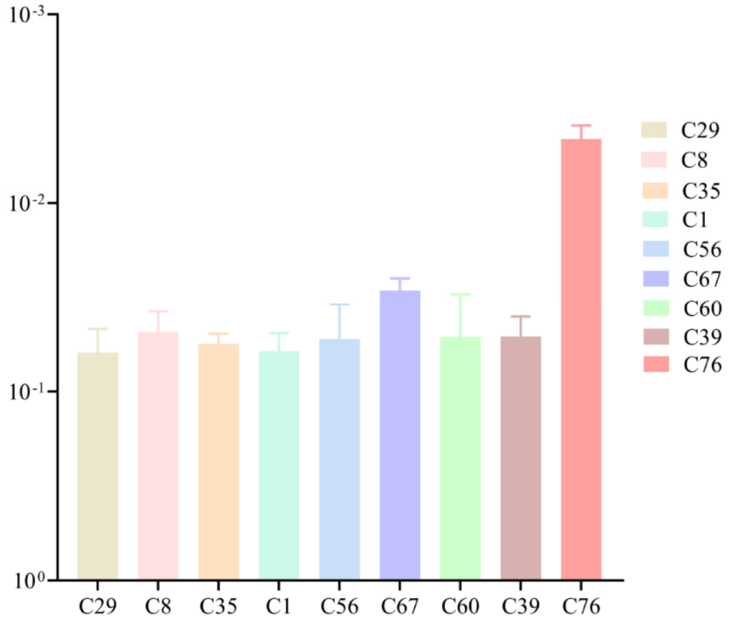

Figure S5. Transconjugative frequencies of *bla*$_{NDM}$-IncX3 and *bla*$_{NDM}$-IncF Ⅱ plasmids.

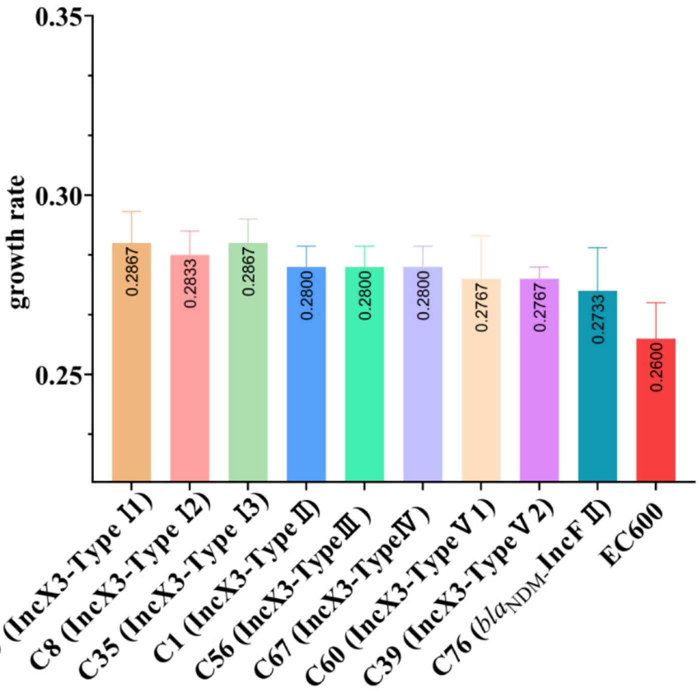

Figure S6. Growth rate of 9 kinds of transconjugants.

A

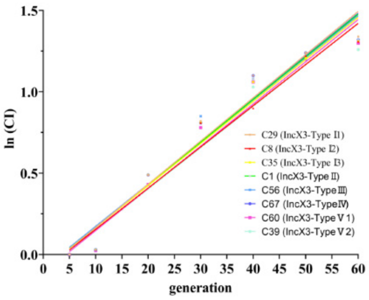

Transconjugant	selection coefficient (*s*)
C29 (IncX3-TypeI1)	0.0244
C8 (IncX3-TypeI2)	0.0239
C35 (IncX3-TypeI3)	0.0237
C1 (IncX3-TypeII)	0.0241
C56 (IncX3-TypeIII)	0.0235
C67 (IncX3-TypeIV)	0.0242
C60 (IncX3-TypeV1)	0.0241
C39 (IncX3-TypeV2)	0.0231

B

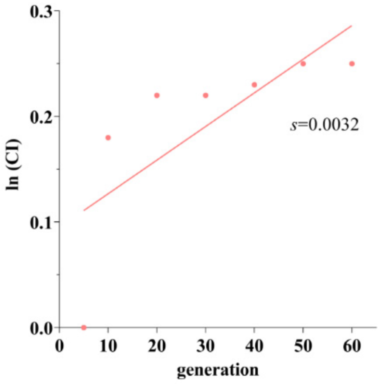

Figure S7. Selection coefficient of 9 kinds of transconjugants. (A) Selection coefficient of carrying *bla*$_{NDM}$-IncX3 transconjugants. (B) Selection coefficient of carrying *bla*$_{NDM}$-IncF II transconjugant.
